# A parallel classification strategy to simultaneous control elbow, wrist, and hand movements

**DOI:** 10.1186/s12984-022-00982-z

**Published:** 2022-01-28

**Authors:** Francesca Leone, Cosimo Gentile, Francesca Cordella, Emanuele Gruppioni, Eugenio Guglielmelli, Loredana Zollo

**Affiliations:** 1 Unit of Advanced Robotics and Human-Centred Technologies, Università Campus Bio-Medico di Roma, Rome, Italy; 2 INAIL Prosthetic Center, Vigorso di Budrio, Italy

**Keywords:** Upper limb, Prosthetic control, Real-time and offline performance, Multi-DoFs control, Prosthetic control, Pattern recognition

## Abstract

**Background:**

In the field of myoelectric control systems, pattern recognition (PR) algorithms have become always more interesting for predicting complex electromyography patterns involving movements with more than 2 Degrees of Freedom (DoFs). The majority of classification strategies, used for the prosthetic control, are based on single, hierarchical and parallel linear discriminant analysis (LDA) classifiers able to discriminate up to 19 wrist/hand gestures (in the 3-DoFs case), considering both combined and discrete motions. However, these strategies were introduced to simultaneously classify only 2 DoFs and their use is limited by the lack of online performance measures. This study introduces a novel classification strategy based on the Logistic Regression (LR) algorithm with regularization parameter to provide simultaneous classification of 3 DoFs motion classes.

**Methods:**

The parallel PR-based strategy was tested on 15 healthy subjects, by using only six surface EMG sensors. Twenty-seven discrete and complex elbow, hand and wrist motions were classified by keeping the number of electromyographic (EMG) electrodes to a bare minimum and the classification error rate under 10 %. To this purpose, the parallel classification strategy was implemented by using three classifiers one for each DoF: the “Elbow classifier”, the “Wrist classifier”, and the “Hand classifier” provided the simultaneous control of the elbow, hand, and wrist joints, respectively.

**Results:**

Both the offline and real-time performance metrics were evaluated and compared with the LDA parallel classification results. The real-time recognition results were statistically better with the LR classifier with respect to the LDA classifier, for all motion classes (elbow, hand and wrist).

**Conclusions:**

In this paper, a novel parallel PR-based strategy was proposed for classifying up to 3 DoFs: three joint classifiers were employed simultaneously for classifying 27 motion classes related to the elbow, wrist, and hand and promising results were obtained.

## Introduction

Myoelectric control systems have been extensively used over the years to restore for upper-limb amputees most of the movements usual in daily living activities [1]. Several solutions based on proportional control, on-off control, finite state machine (FSM), and pattern recognition (PR) control have been previously investigated to restore the lost functionality of the arm [2].Typically, surface electromyographic signals (sEMG) are widely considered the best non-invasive representation of muscular activity [3] and a natural interface to control both the prosthetic devices and the virtual reality prosthesis during real-time interactive tasks, in a non-invasive way [4]. The simultaneous control of combined movements of different joints (e.g. pouring water into a glass) ensures greater dexterity than the sequential one. The simultaneous multi-DoFs control can be easier to implement and natural by using PR strategies instead of conventional myoelectric control systems, as the ON/OFF control and the proportional one [5]. This latter consider only the amplitude of the EMG signal for specific control sites [6, 7].

The most employed PR algorithms for the classification of discrete and combined movements for two and three DoFs are: Linear Discriminant Analysis (LDA) [8], Support Vector Machines (SVM) [9], Artificial Neural Networks (ANN) [10], Wavelet Neural Network (WNN) [11] and some deep learning methods based on decoding user’s intention through sEMG signals [12]. In particular, the simultaneous control of different joints is considered a needed capability to restore upper limb functionality, especially for transhumeral and shoulder disarticulation amputees who have undergone Targeted Muscle Reinnervation (TMR) surgery [13].

However, a limitation of the proposed strategies is the managing of one DoF by the user during complex multi-DoFs tasks. Such sequential control generates unnatural movements that required also a cognitive burden in planning the intended movement because the user cannot perform fluid, lifelike combined movements [14].

The following articles have been found in the literature in which the simultaneous control of different joints have been employed. Technological solutions based on high-density EMG signals [15], neural firing rates, and intramuscular EMG recordings [16] have been proposed to control from three up to six DoFs. In detail, in [15], the EMG-driven large scale model has been used to estimate wrist-hand musculoskeletal function, by considering motion capture data and eight pairs of disposable bipolar EMG electrodes. This system allowed to control, without using a PR-based approach, a total of three DoFs including forearm pronation/supination, wrist flexion/extension and hand opening/closing. In [16], a modified (in terms of thresholds and non-unity gains) Kalman filter (MKF) was used to control over six DoFs DEKA “LUKE” arm by extracting motor intent from the neural firing rates and the mean absolute value of intramuscular EMG recordings. The considered DoFs were related to flexion/extension and abduction/adduction of the thumb; flexion/extension of the index finger; flexion/extension of the middle finger; and flexion/extension and pronation/ supination of the wrist. In [17], 6 EMG channels were used to perform three bidirectional movements simultaneously: 6 phantom wrist and hand movements (finger flexion, finger extension, pronation and supination of the stump, wrist flexion and wrist extension) were discriminated by separating classes with hyperplanes computed with the Lawrence method [18, 19]. More recent studies [20] have introduced PR-based simultaneous control strategies for managing up to 2–3 DoFs related to different joints like the elbow, the wrist, and the hand [7, 21, 22]. In particular, three main strategies have been proposed in the literature to apply pattern recognition strategies to both discrete and combined movements. The first strategy trained a single LDA classifier by labeling as unique classes both discrete (1 DoF) and combined movements that involved the activation of two joints [23]; the second strategy introduced three single LDA classifiers that predicted the simultaneous movement of three fingers in a non-human primate, by applying a parallel classification scheme [24]; the third one presented a control strategy for the classification of simultaneous movements of wrist and hand joints, (named conditional parallel classification strategy) based on three parallel LDA classifiers that employed conditional probability to define the boundaries between similar classes of movement [25]. Others studies as [14] have employed hierarchical and parallel classification strategies based on LDA classifiers in order to discriminate both discrete and combined motions as separate classes. The considered motions were: hand open/close (HO/HC), wrist extension/flexion (WE/WF), wrist supination/pronation (WS/WP), elbow extension/flexion (EE/EF), no motion (NM) and all 2-DoFs combined motions. The hierarchical strategy obtained the best performance from 6 healthy control subjects, by keeping below 15 % the classification errors.

To improve the classification performance when considering combined wrist/hand classification tasks, the use of intramuscular EMG was investigated on two PR methods [26]: the first was based on a single classifier that discriminated between 1 DoF and 2 DoFs motion classes; the second method employed a parallel set of three classifiers to predict up to 3 DoFs. The results showed that the classification error significantly decreased when using the intramuscular EMG compared to surface EMG for the parallel configuration (p < 0.01), but not for the single classifier. Moreover, most of the studies have considered only the offline performance metrics, without testing the introduced classification strategy also in real-time. This may be a relevant limitation for the prosthetic control assessment, since many studies have shown that offline accuracy does not necessarily correspond to real-time performance [1, 27].

In this paper, a novel parallel PR-based strategy was proposed for classifying up to 3 DoFs. The novelty consists of using an effective dataset organization that aims to train three joint classifiers for classifying 27 motion classes related to the elbow, wrist, and hand. To this purpose, both the offline and real-time performances were introduced to assess the robustness of the parallel PR-based strategy. In detail, it relies on the use of three parallel LR classifiers with the regularization parameter and the features extraction (FE) step to simultaneously control multiple DoFs related to the elbow, wrist and hand joints. We extracted five time-domain features from the raw sEMG signals of 6 sEMG sensors and 26 motion classes (6 discrete motions and 20 combined motions) were discriminated. The analysis of the real-time performance was evaluated by means of the motion selection time, motion completion time, and completion rate for all the 27 motion classes [28]. The offline and real-time performances were compared with the LDA benchmark algorithm, by using three LDA classifiers with the same feature extraction and parallel classification strategy. To date, the single, hierarchical, and parallel classification strategies, based on the LDA classifiers, were introduced to discriminate 3 DoFs but related only to wrist and hand joints ( for an amount of 19 motion classes).

The structure of the paper is as follows: " Materials and methods" section introduces the algorithm and the classifier strategy with the parameters selected to obtain the best performance; " Parallel classification strategy" section reports the results in terms of F1Score obtained from 15 healthy subjects; " Results" section analyzes and discusses the results and draws the conclusion.

## Materials and methods

### Logistic regression algorithm

The adopted Logistic regression model used the following logistic function to evaluate the class membership probability (Eq. 1):

1 $$\begin{aligned} P(1\mid {x},\theta )={\left\{ \begin{array}{ll}g(\theta ^T\cdot {x})=\frac{1}{1 + e^{-\theta ^T\cdot {x}+\theta _0}} \\ 1-P(y = 0\mid {x},\theta )\end{array}\right. } \end{aligned}$$

where $\theta$ and $\theta _0$ are the classification parameters vector and bias term, respectively, while $g(\cdot {})$ is the logistic function.

The following cross-entropy error cost function was adopted with a regularization term to improve the generalization performance on unseen data Eq. 2:

2 $$\begin{aligned} \begin{array}{l} J(w)=\sum \limits _{i = 1}^{n}-y^{(i)}\cdot {\ln {g(\theta ^T\cdot {x^{(i)}}+\theta _0)}}-(1-y^{(i)})\\ \cdot {\ln {(1-g(\theta ^T\cdot {x^{(i)}}+\theta _0)}}) +\frac{\lambda }{2}\Vert w\Vert ^2 \end{array} \end{aligned}$$

where *m* is the number of samples belonging to the TrainingSet and $y^{(i)}$ is the known class membership of the *i*-th sample, and $\lambda$ is the regularization parameter that adds penalty on the cost function when the magnitudes of the fitting parameters increase. The gradient of the cost function is a vector whose the $j^{(th)}$ element is defined as follows (Eq. 3):

3 $$\begin{aligned} {\left\{ \begin{array}{ll} \frac{\partial J(\theta )}{\partial \theta _0}=\frac{1}{m}\sum \limits _{i = 1}^{m}(h_\theta (x^{(i)})-y^{(i)})x_j^{(i)} &{} \text {for j = 0} \\ \frac{\partial J(\theta )}{\partial \theta _0}=\frac{1}{m}\sum \limits _{i = 1}^{m}(h_\theta (x^{(i)})-y^{(i)})x_j^{(i)}+ \frac{\lambda }{m}\theta _j &{} \text {for j}\ge \text {0} \end{array}\right. } \end{aligned}$$

The first-order iterative optimization algorithm “Gradient descent” was used for finding a local minimum of the multivariate differentiable cost function, with a maximum number of iterations equal to 150 [29]. In particular, the Polack-Ribiere flavour of conjugate gradients was used to compute search directions; a line search with quadratic and cubic polynomial approximations and the Wolfe-Powell stopping criterion was employed together with the slope ratio method for guessing initial step sizes.

The prediction of class labels $h_\theta$ for the LR algorithm was achieved by comparing the probability distribution *P*(*y*|*x*) with the decision threshold (TH) defined in Eq. 4:

4 $$\begin{aligned} h_\theta ={\left\{ \begin{array}{ll}P(1\mid {x},\theta )\ge {TH}\rightarrow {1} \\ P(1\mid {x},\theta )<{TH}\rightarrow {0}\end{array}\right. } \end{aligned}$$

### Linear discriminant analysis

Three LDA classifiers, with the five time domain features previously introduced, were employed in order to make a comparison with the performance of the three LR classifiers. The training of the classifiers was performed by using Eqs. 5 and 6. In detail, the LDA is a binary supervised machine learning algorithm that guarantees the maximum class separability [30], by transforming the features into a lower dimensional space that maximizes the ratio of the between-class variance to the within-class variance.

The following decision function was used to discriminate between two different classes and to assign class label 1 or 2 to unknown data (Eq. 5):

5 $$\begin{aligned} h_\beta (x)={\left\{ \begin{array}{ll}(\beta ^T\cdot {x}+\beta _0)\ge {0}\rightarrow {1} \\ (\beta ^T\cdot {x}+\beta _0)<{0}\rightarrow {2}\end{array}\right. } \end{aligned}$$

where $\beta$ and $\beta _0$ are, respectively, the classification parameters vector and the bias term. The classification parameters can be evaluated as follows (Eq. 6):

6 $$\begin{aligned} {\left\{ \begin{array}{ll}\beta =\Sigma ^{-1}\cdot (\mu _1-\mu _2)\\ \beta _0=-\beta ^T\cdot \left( \frac{\mu _1+\mu _2}{2} \right) +\ln \left( \frac{\Pi _1}{\Pi _2}\right) \end{array}\right. } \end{aligned}$$

where $\Sigma$ is the pooled covariance matrix, and $\mu _1$, $\mu _2$, $\Pi _1$, $\Pi _2$ are the mean vectors and the prior probabilities of class 1 and class 2, respectively. In order to solve the multi-class classification problem with a binary algorithm as the LDA, a *one vs. all* strategy was implemented.

The class label (c) was predicted according to Eq. (7):

7 $$\begin{aligned} \begin{array}{l} h_\beta (x)=\max _{c}\left( {}_c\beta ^T\cdot {x}+{}_c\beta _0 \right) \\ {\left\{ \begin{array}{ll}{}_c\beta =\Sigma ^{-1}\cdot (\mu _c)\\ {}_c\beta _0=-{}_c\beta ^T\cdot \left( \frac{\mu _c}{2} \right) +\ln \left( \Pi _c\right) \end{array}\right. } \end{array} \end{aligned}$$

where ${}_c\beta$ and ${}_c\beta _0$ are the classification parameter vector and the bias term of c class, respectively. An ad-hoc developed software was implemented in Matlab for the construction of the three LDA classifiers.

The performance were evaluated offline through F1Score and the misclassification error values, while, in real-time, motion selection time, motion completion time and motion completion rate were used. The Mann–Whitney test (U-test) at p < 0.05 has been employed for comparing LR and LDA classifiers in common datasets [31] for both offline and real-time evaluation.

### Features extraction

In this study, feature extraction was performed by calculating the following five time domain features [32]: Enhanced Mean Absolute value (EMAV), Enhanced Wavelength (EWL), Slope Sign Change (SSC), Root Mean Square (RMS), Variance (VAR). Data were segmented by using a windows of 150 ms with an overlap of 100 ms [33]. In short, the EMAV is an extension of the MAV that is defined as the summation of absolute values of EMG signals [34] and can be calculated by Eq. 8:

8 $$\begin{aligned} \begin{array}{l} EMAV_i=\frac{1}{L}\sum \limits _{i = 1}^{L}\left| (x_i)^p|\right| \\ p={\left\{ \begin{array}{ll} 0.75, \quad if \; i \; \ge \; 0.2 \; L \; \& \; \; i \; \le \; 0.8 \; L \\ 0.50, otherwise\end{array}\right. } \end{array} \end{aligned}$$

The EWL is an extension of WL that represents the cumulative length of the EMG signal waveform and can be calculated as Eq. 9:

9 $$\begin{aligned} \begin{array}{l} EWL=\sum \limits _{i = 2}^{L}\left| (x_i-x_{i-1})^p\right| \\ p={\left\{ \begin{array}{ll} 0.75, \quad if \; i \; \ge \; 0.2 \; L \; \& \; \; i \; \le \; 0.8 \; L \\ 0.50, otherwise\end{array}\right. } \end{array} \end{aligned}$$

where in both the Eqs. 8, 9, $x_i$ is the EMG data and L is the number of samples in each time window and the parameter *p* is used to enhance the information content at the middle region of the time window [32].

The Slope sign change represents the number of times the slope of EMG signal changes sign and it is defined as in Eq. 10:

10 $$\begin{aligned} \begin{array}{l} SSC=\frac{1}{L}\sum \limits _{i = 2}^{L-1}f[(x_i-x_{i-_1})\times (x_i-x_{i + 1})] \\ f(x_i)={\left\{ \begin{array}{ll} 1, &{} x \ge threshold \\ 0, &{} otherwise \end{array}\right. } \end{array} \end{aligned}$$

The Root Mean Square is the mean power of the signal and is defined by Eq. 11:

11 $$\begin{aligned} RMS=\sqrt{\frac{1}{L}\sum \limits _{i = 1}^{L}x_i^2} \end{aligned}$$

The Variance represents a statistical measure of how signal varies from its average value and is defined by Eq. 12:

12 $$\begin{aligned} VAR=\frac{1}{L-1}\sum \limits _{i = 1}^{L}x_i^2 \end{aligned}$$

### Experimental setup and protocol

Fifteen healthy people (aged: $36 \pm 13$), were enrolled in the study. The sEMG data were acquired at 1 kHz by using the DAQ USB 6002 device and a suitable software on the Labview platform. The PC (Samsung Intel(R) Core (TM) i7–4500U CPU @ 1.80 GHz) and DAQ communicated by means of an USB port. Six commercial active sEMG sensors (Ottobock 13E200 = 50, 27 mm 18 mm 9.5 mm) were placed on the subject arm: four sensors were equidistantly fixed on an elastic bracelet placed about 4 cm below the subject’s elbow Fig.A 1; instead the remaining two sensors were used to include biceps and triceps activity to record the elbow flexion and extension, respectively. The number of sEMG sensors was chosen equal to six to avoid a high-dimensional feature space and maintain simple the hardware [35].

**Fig. 1:**
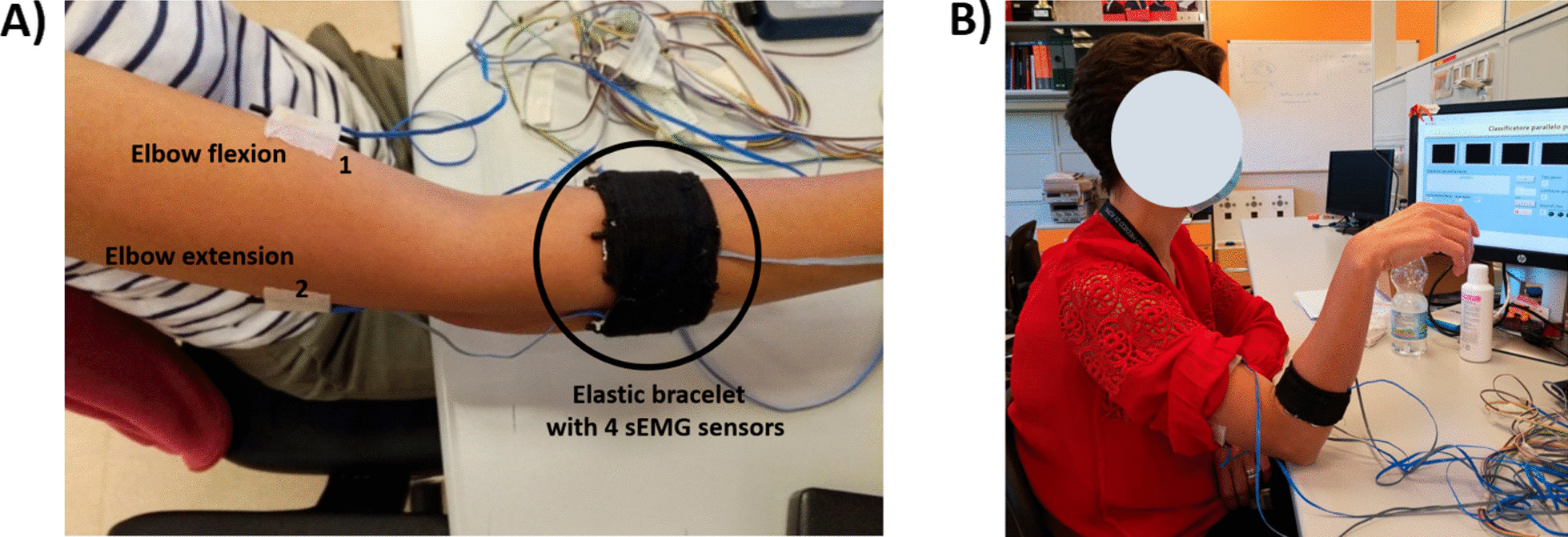
The experimental setup was composed of: (i) a sEMG elastic bracelet, (ii) NI DAQ USB 6002, (iii) Labview interface software to acquire the sEMG signals.Written informed consent for the publication of this image was obtained

The subject was sitting in front of a monitor Fig. 1B and was asked to produce each of the 27 gestures reported in Table 1: the discrete motions were elbow flexion and extension, hand open and close and, wrist supination and pronation; the combined motions of each joint classifier (elbow, wrist, and hand) involve from two DoFs up three DoFs. The participants were asked to produce each of these gestures for four times and to hold it for 3 s with an interval of about 2 s rest state between each repetition, to collect data for the DataSet. In detail, the sEMG data were organized in a DataSet matrix with 6 columns, each corresponding to an EMG sensor. The training and test sets were obtained by using the *two ways data split approach* [36] for both the algorithms: 70 % of the data were reserved for the *“TrainingSet”* (TR), while the remaining 30 % of the data for the *“Test Set”* (TS). The one vs. all approach was employed to adapt the LDA and LR classification algorithms to the multi-class classification problem. For the LR algorithm, the first-order iterative optimization algorithm “Gradient descent” was used to set the optimal internal parameters.

**Table 1:** Report of daily activities where the considered 27 motion classes are involved

**Motion Classes**		**Motion Classes**	***Daily Activity***
Discrete	1	F	Elbow flexion for bringing something to you
	2	E	Elbow extension for giving something to someone
	3	C	Close hand for holding something
	4	O	Open hand for giving something
	5	S	Wrist supination for rotating an object
	6	P	Wrist supination for rotating an object
Combined	7	FCS	Brings a biscuit to your mouth
	8	EOP	Rest your open hand on the table
	9	FCP	Bring the back of your closed hand to your forehead
	10	EOS	Bring the back of your open hand onto the table
	11	FOS	Bring your open hand to your mouth
	12	ECP	Pour water into a glass
	13	FOP	Bring the back of your open hand to your forehead
	14	ECS	Bring the back of your closed hand onto the table
	15	CS	Rotate the back of your closed hand to open the lid of a pot
	16	OP	Rotate your open hand down
	17	OS	Rotate the back of your open hand
	18	ES	Turn your closed hand down to turn the door handle
	19	FC	Flex your elbow with your hand closed (bring your fist to your mouth)
	20	EO	Extend your open hand (karate blow)
	21	FO	Flex your elbow with your hand closed
	22	EC	Extend your elbow with your hand closed
	23	FS	Flex your elbow and rotate your wrist upwards
	24	EP	Extend your elbow and rotate your wrist down
	25	FP	Flex your elbow and rotate your wrist down
	26	CP	Close the door

The structure of the parallel classification strategy, implemented with both LR and LDA three classifiers, will be discussed in the next section.

## Parallel classification strategy

The parallel classification strategy, introduced in this section, was implemented by using three classifiers one for each DoF: the “Elbow classifier”, the “Wrist classifier”, and the “Hand classifier” provided the simultaneous control of the elbow, hand, and wrist joints, respectively. In detail, the proposed parallel classification strategy was implemented with the LR algorithm, to recognize both discrete and combined elbow, wrist, and hand motions. Then, the same strategy was reproduced using LDA algorithm in order to perform a comparative analysis. The control scheme providing the final decision is composed of the independent outputs of the three joint classifiers.

**Fig. 2:**
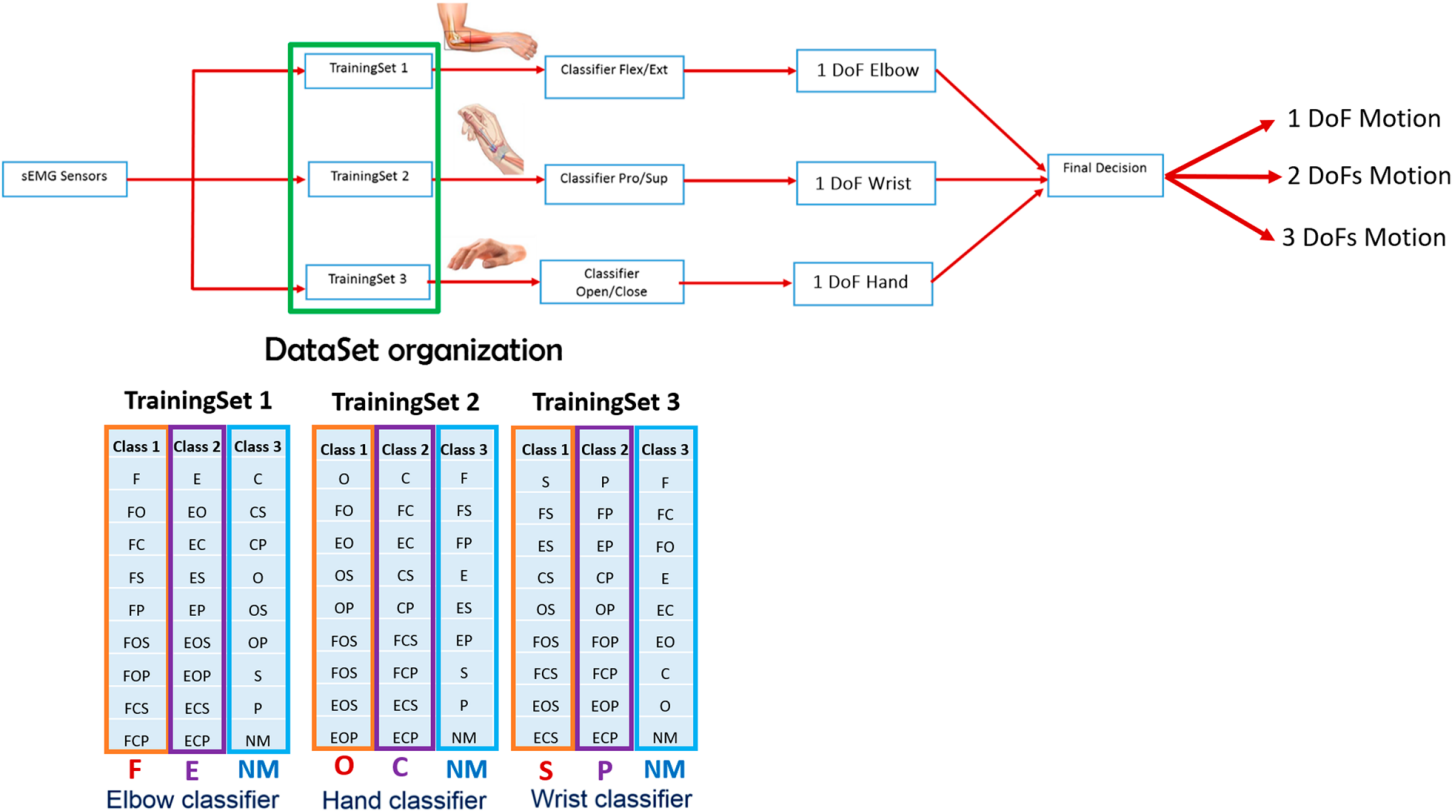
Block diagram describing the parallel classification strategy composed by three joint classifiers (“Elbow classifier”, “Wrist classifier” and “Hand classifier”), each trained with a different TrainingSet. F: elbow flexion; FO: elbow flexion with hand open; FC: elbow flexion with hand close; FS: elbow flexion with wrist supination; FP: elbow flexion with wrist pronation; FOS: elbow flexion with hand open and wrist supination; FOP: elbow flexion with hand open and wrist pronation; FCS: elbow flexion with hand close and wrist supination; FCP: elbow flexion with hand close and wrist pronation; E: elbow extension; EO: elbow extension with hand open; EC: elbow extension with hand close; ES: elbow extension with wrist supination; EP: elbow extension with wrist pronation; EOS: elbow extension with hand open and wrist supination; EOP: elbow extension with hand open and wrist pronation; ECS: elbow extension with hand close and wrist supination; ECP: elbow extension with hand close and wrist pronation; C: hand close; CS: hand close with wrist supination; CP: hand close with wrist pronation; O: hand open; OS: hand open with wrist supination; OP: hand open with wrist pronation; S: wrist supination; P: wrist pronation; NM: no motion class

In particular, the “Elbow classifier” was trained with the TrainingSet 1, organized as reported in Fig. 2: from the DataSet matrix, described above, that contained the recordings of four repetitions of each of the 27 motion classes, the discrete and combined motion classes were labeled into three output classes. In detail, the output of the “Elbow classifier” determines the elbow flexion ( labeled as “Class 1”), extension ( labeled as “Class 2”) and the “other motions” ( labeled as “Class 3”) not involving the use of the elbow. In particular, the “Class 1” was represented by the examples of nine discrete and combined motion classes that involved the elbow flexion. The “Class 2” was represented by the examples of nine discrete and combined motion classes that involved the elbow extension. While the “Class 3” was represented by the examples of nine discrete and combined motion classes that involved other joints, as the hand and wrist. The “Wrist classifier” was trained with the TrainingSet 2 and the same considerations made for the “Elbow classifier” can be applied. The unique difference was the output of the “Wrist classifier”, that determines wrist supination (labeled as “Class 1”), pronation (labeled as “Class 2”) and the “other motions” (labeled as “Class 3”) that does not involve the use of the wrist. Finally, the “Hand classifier” was trained with the TrainingSet 3. The output of the “Hand classifier” manages the hand opening (labeled as “Class 1”), closing (labeled as “Class 2”) and the “other motions” (labeled as “Class 3”) not implying the use of the hand. Thus, for the parallel classification strategy, the performance were evaluated across the three output classes of each joint classifier. The final decision of the parallel classification strategy depends on the simultaneous outputs of the three classifiers Fig. 2: if only one joint classifier outputs the “Class 1” or “Class 2” and the others two classifiers output the “Class 3”, the final output will be a 1 DoF motion class; if two joint classifiers output the “Class 1” or “Class 2” and the other one output the “Class 3”, the final output will be a 2 DoF motion class; finally if all the three classifiers output the the “Class 1” or “Class 2”, the final decision will be a 3 DoFs motion classes. In this way, considering the final output of the parallel classification strategy, the classification of 27 motion classes can be obtained from all the possible combinations of the three outputs of the joint classifiers.

In particular, the proposed model discriminated between the following 26 motion classes and no motion class, for a total of 27 tasks: 6 discrete motions (elbow flexion, elbow extension, hand open, hand close, wrist supination, wrist pronation), the rest state, and other 20 complex motions, performed during daily life activities, derived from the combination of the discrete motions (Table 1).

**Fig. 3:**
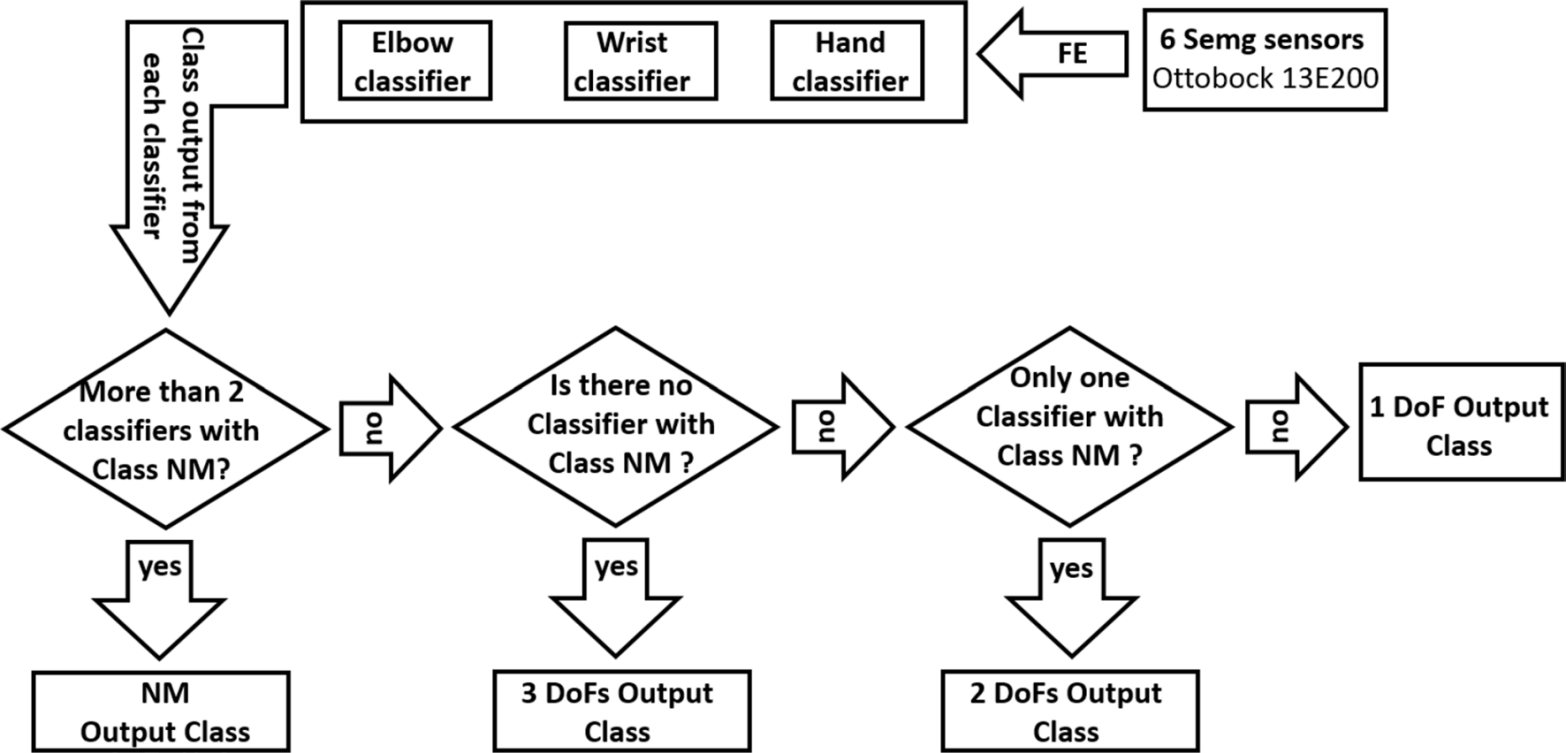
Flowchart for the parallel classification strategy, used for classifying 27 motion classes related to elbow, wrist and hand joints

In this study, all the six EMG channels were always used for every classification decision. The classification scheme is illustrated in Fig. 3, by means of a flowchart: firstly, the features extraction (FE) was performed from the acquisition of 6 sEMG sensors Ottobock 13E200 and the TD features were sent as input for each classifier (LR or LDA). Then, the final output of this strategy is the movement class derived from the output combination of the three classifiers and it can be: no motion output class if more than two classifiers output the “Class 3”; a 3 DoFs output class if none of the classifiers output “Class 3”; a 2 DoFs output class if only one classifier output “Class 3”; a 1 DoF motion class if two classifiers output “Class 3”;

## Results

### Offline performance

**Fig. 4:**
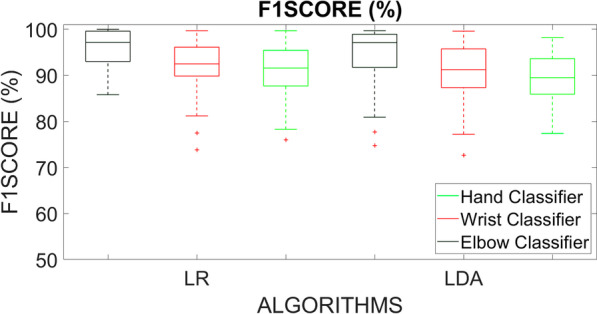
Box plots of the average F1Score values calculated on 15 healthy subjects using LR and LDA algorithms with five time domain features, tested on “TS,” for the Elbow, Wrist, and Hand classifiers

The offline results of the parallel classification strategy are reported for both LR and LDA algorithms in Table 2, Table 3, Table 4 for the three classifiers (“Elbow classifier”, “Wrist classifier”, and “Hand classifier”) in terms of F1Score (Fig. 4).

**Table 2:** F1Score values for the “Elbow classifier” obtained with LR and LDA algorithms

	**ELBOW CLASSIFIER**	
	**F1Score**	
	**LR**	**LDA**
CLASS 1	$95,0 \pm 4,5$	$93,1 \pm 7,3$
CLASS 2	98,6 ± 2,3	$97,7 \pm 2,9$
CLASS 3	94,6 ± 4,1	92,7 ± 6,5
MEAN	96,1 ± 2,9	94,5 ± 4,8

F: elbow flexion, FO: elbow flexion with hand open. FC: elbow flexion with hand close, FS: elbow flexion with wrist supination, FP: elbow flexion with wrist pronation, FOS: elbow flexion with hand open and wrist supination, FOP: elbow flexion with hand open and wrist pronation, FCS: elbow flexion with hand close and wrist supination, FCP: elbow flexion with hand close and wrist pronation, E: elbow extension, EO: elbow extension with hand open, EC: elbow extension with hand close, ES: elbow extension with wrist supination, EP: elbow extension with wrist pronation, EOS: elbow extension with hand open and wrist supination, EOP: elbow extension with hand open and wrist pronation, ECS: elbow extension with hand close and wrist supination, ECP: elbow extension with hand close and wrist pronation; C: hand close, CS: hand close with wrist supination, CP: hand close with wrist pronation, O: hand open, OS: hand open with wrist supination, OP: hand open with wrist pronation, S: wrist supination, P: wrist pronation, NM: no motion class

**Table 3:** F1Score values for the “Wrist classifier” obtained with LR and LDA algorithms

	WRIST CLASSIFIER	
	F1Score	
	LR	**LDA**
CLASS 1	$91,9 \pm 4,3$	$91,0 \pm 5,1$
CLASS 2	$94,9 \pm 5,0$	$94,4 \pm 5,2$
CLASS 3	$88,3 \pm 6,3$	$86,8 \pm 6,3$
MEAN	$91,7 \pm 4,1$	$90,7 \pm 4,1$

The reported results were obtained by considering the mean values on 15 healthy subjects and by segmenting data with a window of 150 ms and an overlap of 100 ms [33] for the feature extraction, as described above, for both the algorithms. The mean F1Score values, over the three output classes, reached 96.1 % ± 2.9 (Table 2), 91.7 % ± 4.1 (Table 3), 91.0 % ± 4.8 (Table 4), for the LR “Elbow classifier”, “Wrist classifier”, and “Hand classifier”, respectively. The mean misclassification error rates remained under 10 %, a value that can be considered acceptable for a system of practical use [4].

**Fig. 5:**
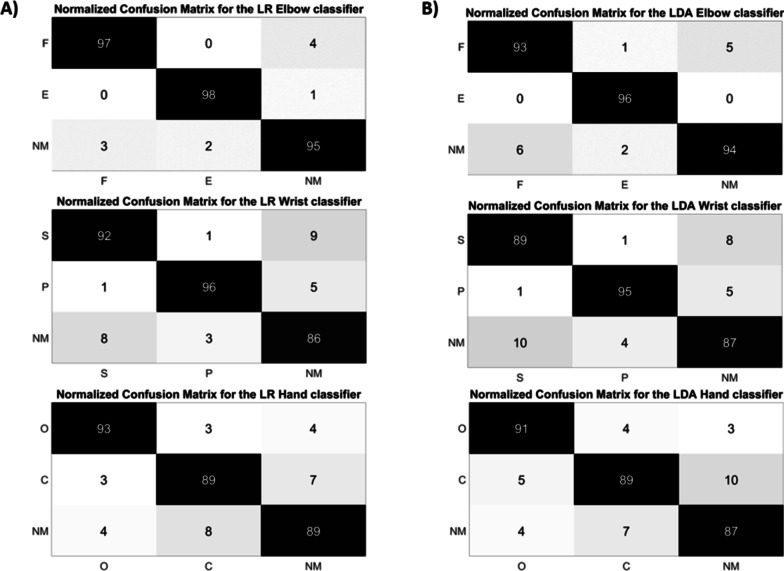
Normalized confusion matrix of the Elbow, Wrist, and Hand Classifiers obtained with the LR (**A**) and LDA (**B**) algorithms. The confusion matrices are normalized concerning the number of data belonging to the “TS”. On the main diagonal the cardinality of the correct classifications is reported; in the top left dial and bottom right dial, the cardinality of the misclassified data, related to the 3 output classes representing the motion DoFs of each joint classifier, are reported

For the LDA “Elbow classifier”, “Wrist classifier”, and “Hand classifier”, the mean F1Score values were equal to 94.5 % ± 4.8 (Table 2), 90.7 % ± 4.1 (Table 3), 89.3 % ± 4.8 (Table 4), respectively. Figure 5 shows also the average confusion matrix when testing both the LR and LDA classifiers on the Test Set, over the three output classes that represent the controllable DoFs of each classifier.

**Table 4:** F1Score values for the “Hand classifier” obtained with LR and LDA algorithms

	HAND CLASSIFIER	
	F1Score	
	LR	**LDA**
CLASS 1	$93,6 \pm 5,4$	$92,3 \pm 4,9$
CLASS 2	$89,7 \pm 5,7$	$87,8 \pm 5,8$
CLASS 3	$89,7 \pm 5,3$	$87,7 \pm 5,4$
MEAN	$91,0 \pm 4,8$	$89,3 \pm 4,8$

**Table 5:** The mean “MST”, “MCT” and “MCR” values related to 1 DoF, 2 DoFs and 3 DoFs, for the LR and LDA algorithms

Motion Classes		**LR**			**LDA**		
MEAN		**MST**	**MCT**	**MCR**	**MST**	**MCT**	**MCR**
1 DoF	F	$0.23 \pm 0.23$	$1.80 \pm 1.37$	$94 \pm 24$	$0.13 \pm 0.15$	$2.04 \pm 1.72$	$90 \pm 21$
	E	$0.55 \pm 0.66$	$2.87 \pm 1.92$	$79 \pm 40$	$0.58 \pm 0.68$	$2.57 \pm 1.95$	$80 \pm 37$
	C	$0.67 \pm 1.50$	$1.56 \pm 1.29$	$94 \pm 24$	$0.62 \pm 1.58$	$1.52 \pm 1.36$	$93 \pm 26$
	O	$0.21 \pm 0.19$	$1.46 \pm 0.99$	$97 \pm 12$	$0.27 \pm 0.52$	$1.83 \pm 1.87$	$87 \pm 30$
	S	$0.17 \pm 0.15$	$1.37 \pm 0.58$	$97 \pm 12$	$1.17 \pm 2.09$	$3.27 \pm 2.43$	$83 \pm 31$
	P	$0.26 \pm 0.19$	$1.32 \pm 0.48$	$100 \pm 0$	$0.26 \pm 0.35$	$1.86 \pm 1.62$	$90 \pm 28$
	R	$0.05 \pm 0.01$	$0.78 \pm 0.77$	$100 \pm 0$	$0.05 \pm 0.01$	$0.80 \pm 0.11$	$100 \pm 0$
	MEAN	** 0.30 ** ± ** 0.42**	** 1.59 ** ± ** 1.05**	** 94.42 ** ± ** 16**	**0.44 ** ± ** 0.76**	**1.98 ** ± ** 1.58**	** 89 ** ± ** 24.71**
2 DoFs	FO	$0.31 \pm 0.20$	$1.53 \pm 0.81$	$94 \pm 24$	$0.30 \pm 0.33$	$1.93 \pm 1.58$	$90 \pm 28$
	FC	$0.40 \pm 0.27$	$1.65 \pm 0.74$	$94 \pm 24$	$0.36 \pm 0.37$	$2.28 \pm 1.76$	$80 \pm 37$
	FS	$0.64 \pm 1.51$	$1.81 \pm 1.30$	$94 \pm 24$	$0.80 \pm 1.61$	$3.05 \pm 2.23$	$70 \pm 41$
	FP	$0.62 \pm 0.61$	$2.05 \pm 1.89$	$91 \pm 26$	$0.80 \pm 1.11$	$2.85 \pm 2.08$	$73 \pm 32$
	EO	$0.55 \pm 0.49$	$2.89 \pm 2.01$	$76 \pm 44$	$0.44 \pm 0.43$	$2.84 \pm 2.05$	$73 \pm 32$
	EC	$0.60 \pm 0.97$	$2.21 \pm 1.89$	$85 \pm 34$	$1.05 \pm 1.81$	$2.98 \pm 2.33$	$77 \pm 45$
	ES	$0.35 \pm 0.27$	$1.88 \pm 0.84$	$100 \pm 0$	$0.64 \pm 0.96$	$3.40 \pm 2.20$	$87 \pm 45$
	EP	$0.40 \pm 0.37$	$1.70 \pm 1.70$	$91 \pm 26$	$0.37 \pm 0.38$	$2.51 \pm 1.84$	$83 \pm 36$
	**CS**	$0.33 \pm 0.30$	$1.88 \pm 1.36$	$94 \pm 24$	$0.63 \pm 0.85$	$2.65 \pm 1.83$	$87 \pm 42$
	CP	$0.26 \pm 0.16$	$1.65 \pm 0.89$	$100 \pm 0$	$0.71 \pm 1.61$	$3.07 \pm 2.21$	$77 \pm 42$
	OS	$0.48 \pm 0.83$	$2.06 \pm 1.79$	$82 \pm 39$	$1.01 \pm 1.85$	$2.97 \pm 2.32$	$70 \pm 46$
	OP	$0.68 \pm 1.54$	$2.13 \pm 2.03$	$82 \pm 38$	$0.70 \pm 1.64$	$2.57 \pm 2.09$	$80 \pm 32$
	MEAN	**0.46 ** ± ** 0.62**	**1.95 ** ± ** 1.43**	**90.25 ** ± ** 25.25**	**0.65 ** ± ** 1.07**	**2.76 ** ± ** 2.04**	** 79 ** ± ** 39**
3 DoFs	FOS	$0.29 \pm 0.16$	$1.40 \pm 0.59$	$100 \pm 0$	$0.20 \pm 0.15$	$2.45 \pm 2.06$	$80 \pm 37$
	FOP	$0.63 \pm 0.91$	$2.14 \pm 1.77$	$88 \pm 33$	$0.20 \pm 0.15$	$2.11 \pm 1.99$	$77 \pm 37$
	FCS	$0.58 \pm 0.80$	$2.13 \pm 2.01$	$82 \pm 39$	$0.31 \pm 0.43$	$2.61 \pm 2.15$	$73 \pm 42$
	FCP	$0.41 \pm 0.39$	$1.96 \pm 1.65$	$91 \pm 26$	$0.29 \pm 0.32$	$2.66 \pm 2.15$	$73 \pm 42$
	EOS	$0.49 \pm 0.40$	$2.58 \pm 1.44$	$88 \pm 33$	$0.59 \pm 0.67$	$3.47 \pm 1.83$	$73 \pm 37$
	EOP	$0.36 \pm 0.21$	$1.34 \pm 0.35$	$100 \pm 0$	$0.30 \pm 0.22$	$1.61 \pm 1.07$	$97 \pm 13$
	ECS	$0.44 \pm 0.38$	$2.20 \pm 1.32$	$94 \pm 24$	$1.17 \pm 1.84$	$3.33 \pm 2.15$	$83 \pm 32$
	ECP	$0.46 \pm 0.28$	$1.46 \pm 0.55$	$100 \pm 0$	$0.42 \pm 0.32$	$2.08 \pm 1.53$	$87 \pm 30$
	MEAN	**0.45 ** ± ** 0.44**	**1.90 ** ± ** 1.21**	**93 ** ± ** 19**	** 0.43 ** ± ** 0.51**	** 2.54 ** ± ** 1.87**	** 80 ** ± ** 34**
	MEAN	**0.42 ** ± ** 0.52**	**1.84 ** ± ** 1.25**	**92 ** ± ** 21**	**0.54 ** ± ** 0.84**	**2.49 ** ± ** 1.87**	**82 **± **34**

The Mann–Whitney test applied to the F1Score points out a not statistically significant difference between LR and LDA algorithms for the “Elbow classifier”, the “Wrist classifier”, and “Hand classifier” (at p < 0.05 Fig. 4, and $\eta ^{2}$ at 95 % CI). A in-depth statistical analysis is reported in Table 6 where also the effect sizes for non-parametric t-tests was evaluated in terms of $\eta ^{2}$ [37]. This metric is an efficient way to compare the sizes of effects [38].

**Table 6:** Statistical analysis based on Mann–Whitney test and effect size values, interpreting according to [46, 47]

		**Statistical Analysis**		**Effect Size Measure**
		**p value Mann–Whitney (p**<**0.05)**	**normal (Z) statistic**	$\eta ^{2}$(**CI=0.95,** **small effect=0.01** **medium effect=0.06** **large effect=0.14)**
Offline Performance (F1Score)	“Elbow Classifier”	0.20	1.29	0.023
	“Wrist Classifier”	0.40	0.85	0.007
	“Hand Classifier”	0.14	1.47	0.023
Real-Time Performance	MST	0.30	-1.04	0.051
	MCT	0.002	-3.14	0.139
	MCR	0.00001	4.52	0.388

### Real-time performance

Both the LR and LDA classifiers were evaluated in real-time by considering the following performance metrics used in [28]: the motion selection time (MST), the motion completion time (MCT) and the motion completion rate (MCR). Specifically, MST is defined as the time from the onset to the first correct classification (i.e the time taken to successfully select a target movement); MCT is the time from movement onset to the 10th correct classification (i.e the time from the onset to the completion of the intended movement); finally, MCR (“success rate”) is the percentage of successfully completed motions out of the total attempted motions.

In [28], the LDA classifier was used to produce in real-time a new prediction every 100 ms. In our study, both the LR and LDA classifiers were tested in real-time and have produced a new prediction every 90 ms.

In particular, the MST, MCT and MCR values have been reported in Table 5 and are related to the mean value obtained from the 15 healthy subjects and calculated over 2 repetitions of all the 27 motion classes.

The mean MCT values among the 27 motion classes was equals to 1.84 ± 1.25 s and $2.49 \pm 1.87$ s for the LR and LDA algorithms, respectively (Table 5).

The mean MCR calculated with both LR and LDA algorithms for the 15 healthy subjects revealed what are the motion classes more difficult to be performed: if considering the discrete motion classes, the elbow extension had the mean MCR values equal to 79 % ± 40 and 80 % ± 37 for the LR and LDA algorithms, respectively.

Regarding the 2 DoFs motion classes, the LR algorithm had the MCR above 85 %, except for the following complex movements that involved the hand or the elbow joint with the wrist rotations: the elbow extension with hand open (76 % ± 44), the hand open with wrist supination (82 % ± 39), and hand open with wrist pronation (82 % ± 38). Instead for the LDA algorithm, a major number of 2 DoFs motion classes that involved elbow with hand and wrist rotations (9 on a total of 12 motion classes) have the mean MCR that ranged from 73 % to 83 % (Table 5).

Also for the 3 DoF motion classes, the LR algorithm had better performance with respect to the LDA algorithm: for the LR, the mean MCR was above 85 % except for the elbow flexion with hand closed and wrist supination (82 % ± 39). Instead, for the LDA, the mean MCR values ranged from 73 to 83%, except for the elbow extension with hand opened and wrist pronation (97 % ± 13) and elbow extension with hand closed and wrist pronation (87 % ± 30) (Table 5).

The Mann–Whitney test applied to the MST values points out no statistically significant difference (“*”) between LR and LDA algorithms, while a significant difference has been revealed for both MCT and MCR (at p < 0.05 and $\eta ^{2}$ at 95 % CI, in Table 6). Subjective evaluation was obtained for both LR and LDA classifiers through a 5-point Liker scale questionnaire (Table 7), in order to retrieve a subjective user’s evaluation about the parallel PR-based approach. To assess how intuitive and user-friendly the users perceived the PR-based classification strategy during the real-time motion tasks, their answers were mapped into numerical values.

**Table 7:** Questionnaire for a subjective evaluation of the parallel control strategy

Likert Questionnaire		
Q1	Which of the 26 required tasks did you find the most difficult to do?	Multiple choice between the 26 motion tasks
Q2	How intuitive was the use of the control system?	From (non intuitive) to 5 (intuitive)
Q3	The classifier immediately recognized the desired movement	From 1 (few) to 5 (lot)
Q4	Did you feel fatigued after testing all the 27 motion classes in real-time?	From 1 (not fatigued) to 5 (fatigued)
Q5	Not all the required movement classes had the same level of difficulty	From 1 (not agree) to 5 (agree)
Q6	Were you able to perform the required movement naturally?	From 1 (natural) to 5 (not natural)
Q7	Which movements do you think are the most challenging?	Multiple choice between: 1) 1 DoF 2) 2 DoFs 3) 3 DoFs

## Discussion

### Offline performance

The results, obtained from 15 healthy subjects, were summarized in terms of mean F1Score values across the three output classes of each joint classifier (Fig. 3) in Tables 2, 3, 4. The “Elbow Classifier” reached the highest mean F1Score values (96.1 % ± 2.9 with LR algorithm, 94.5 % ± 4.8 with LDA algorithm); the “Wrist Classifier” obtained the mean F1Score values equals to 91.7 % ± 4.1 with LR algorithm and 90.7 % ± 4.1 with LDA algorithm; the “Hand Classifier” reached the lowest values equals to 91.0 % ± 4.8 with LR algorithm, and 89.3 % ± 4.8 with the LDA algorithms. The discrimination of hand motions and combined wrist movements was more difficult also in [25]. These results seem to be very promising if we consider the importance for amputee subjects of controlling simultaneously more than two DoFs during daily living activities.

Confusion matrices, shown in Fig. 5, confirmed the positive results of the accuracy parameter. The cardinality of the correct classifications on the main diagonal underlined the high classification accuracy even if some misclassified data out of the main diagonal suggested a slightly lower performance of both LR and LDA “Wrist classifier” and “Hand classifier” with respect to the “Elbow classifier.” This can be due to the major difficulty to discriminate between combined wrist and hand motion classes.

It is interesting to note that the parallel classification strategy with the three LR classifiers obtained the best offline classification performances both in terms of F1Score. The statistical analysis, based on the Mann–Whitney test, confirmed no statistically significant difference (“*”) between the F1Score values of the LR and LDA “Elbow classifier”, “Wrist classifier”, and “Hand classifier” (at p < 0.05). Small effect sizes were found for the mean F1Score related to “Elbow classifier” and “Hand classifier”, while a medium effect was revealed for the “Wrist classifier” (in Table 6).

### Real-time performance

The real-time results considered the MCT metric to determine the correctness or not of the classification of the 27 motion classes. This performance parameter (MCT) required 10 correct predictions within 5 s to consider a motion completed [28]. An individual motion class can be classified as failed if the MCT is over 5 s.

**Fig. 6:**
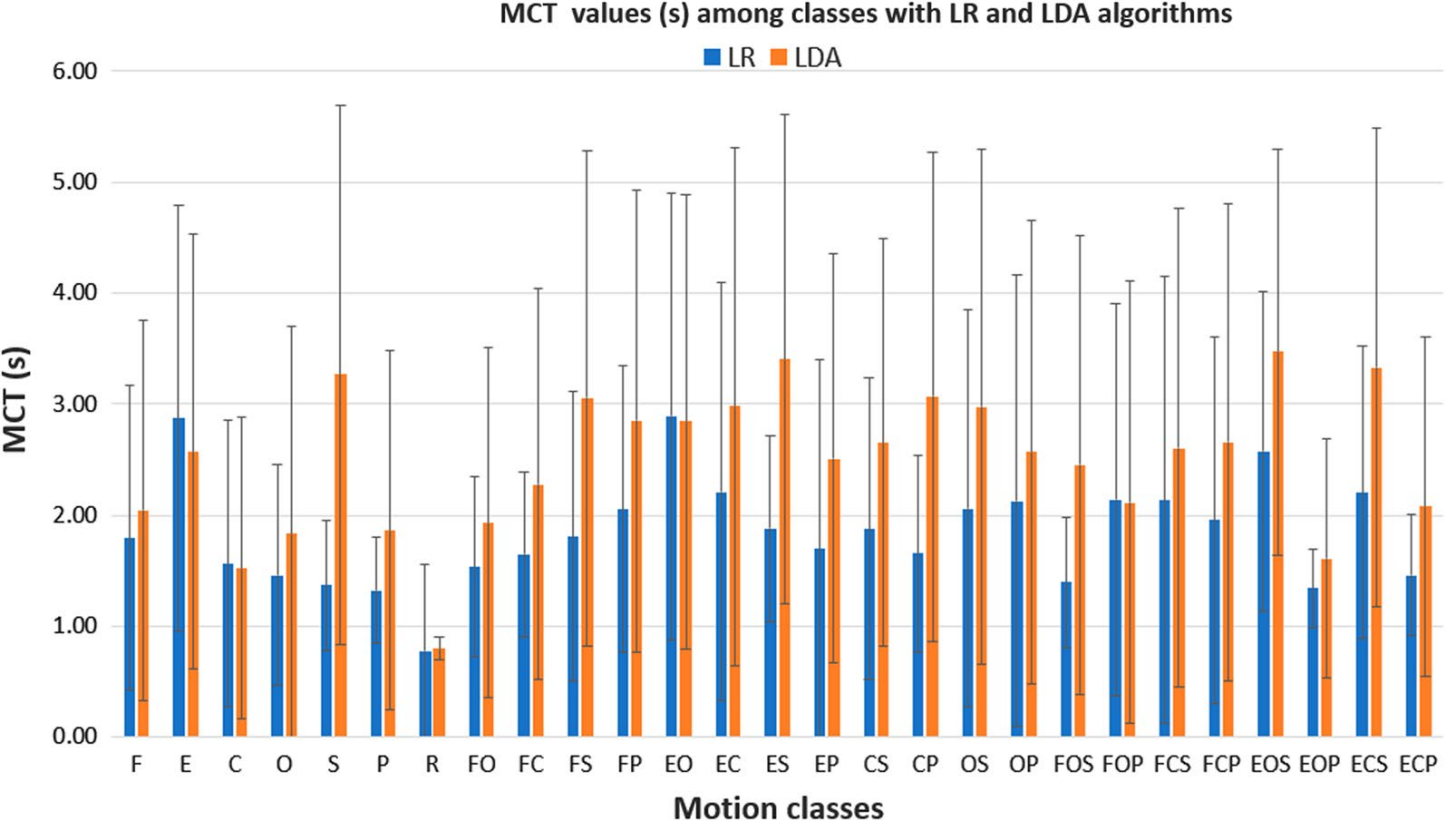
Mean and standard deviation of motion completion time values obtained from the two real-time trials performed by subjects, for all the 27 motion classes, with both LR (blue color) and LDA (orange color) algorithms

The MCT values obtained with both LR and LDA algorithms were reported in Fig. 6: for the LR algorithm, the mean “MCT” values were equal to $1.73 \pm 0.58$ s, $1.95 \pm 0.36$ s, and 1.90 ± 0.45 s for the 1 DoF, 2 DoFs, and 3 DoFs motion classes, respectively.

Conversely, for the LDA algorithm the mean MCT values were equal to $2.18 \pm 0.63$ s, 2.76 ± 0.40 s, and $2.54 \pm 0.63$ s for the 1 DoF, 2 DoFs, and 3 DoFs motion classes, respectively (Fig. 6). The results suggest that the difference in real-time prediction is not marginal: the mean MCT values were over 2 s for the LDA with respect to the LR algorithm (less than 2 s) for 2 and 3 DoFs.

In addition, the LDA classifiers presented the MCT values one second higher than LR classifiers for the following motion classes: supination (S, 1.90 s), elbow flexion with wrist supination (FS, 1.24 s), elbow extension with wrist supination (ES, 1.52 s), hand closing with wrist pronation (CP, 1.41 s), elbow flexion with hand opening and wrist supination (FOS, 1.05 s), elbow extension with hand closing and wrist supination (ECS, 1.13 s) (Fig. 6). The questionnaire confirmed that subjects encountered difficulty to perform the motion tasks that resulted with the highest MCT and lowest MCR values (Fig. 9). In fact, looking at the answers to Q2, the subjects found more intuitive the control based on LR algorithm than the one based on LDA (Fig. 9). Also, according to the answers to Q3, the subjects perceived the classifier with LR as more ready and robust than with LDA. Finally, when the control was based on LDA classifiers, the 2 and 3 DoFs motion tasks were considered more difficult than LR-based control (according to the answers to Q4) (Fig. 9).

**Fig. 7:**
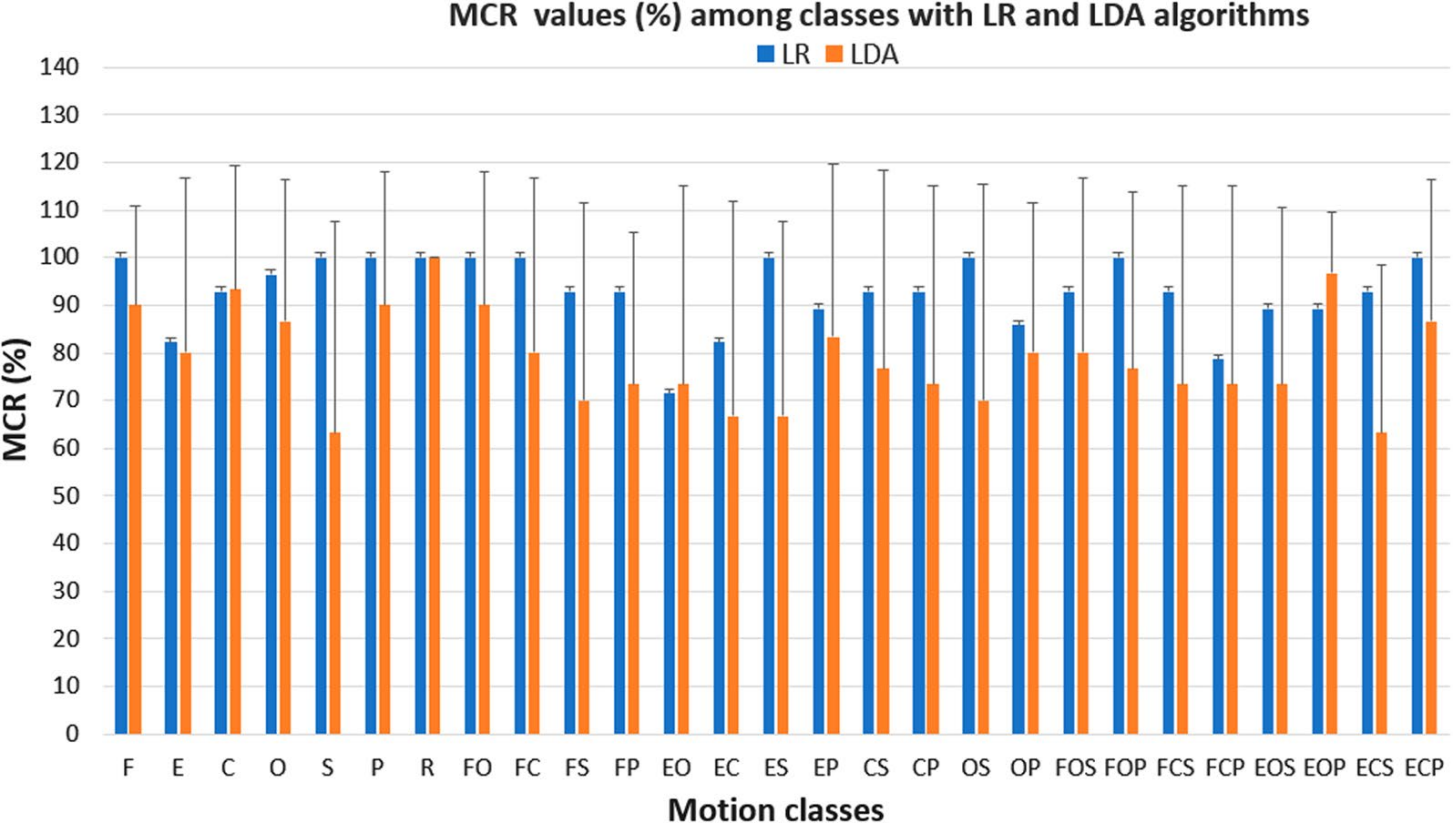
Mean and standard deviation of motion completion rate values obtained from the two real-time trials performed by subjects, for all the 27 motion classes, with both LR (blue color) and LDA (orange color) algorithms

**Fig. 8:**
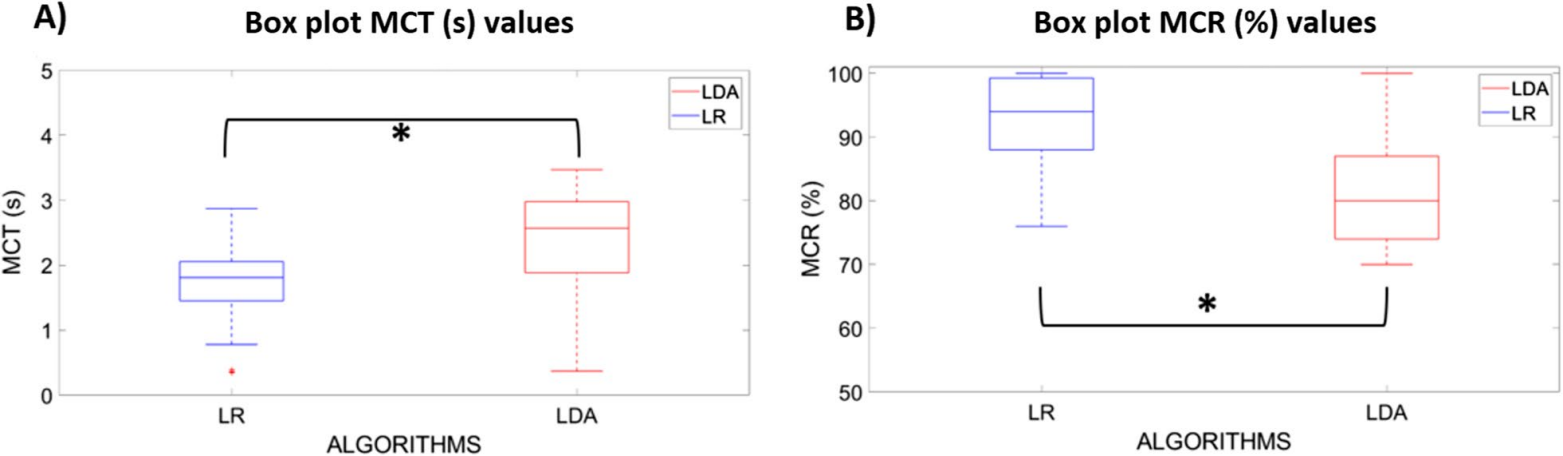
**A** Box plot of the average MCT values calculated on 15 healthy subjects using LR and LDA algorithms. **B** Box plot of the average MCR values calculated on 15 healthy subjects using LR and LDA algorithms. Statistical significance is indicated by “*”

**Fig. 9:**
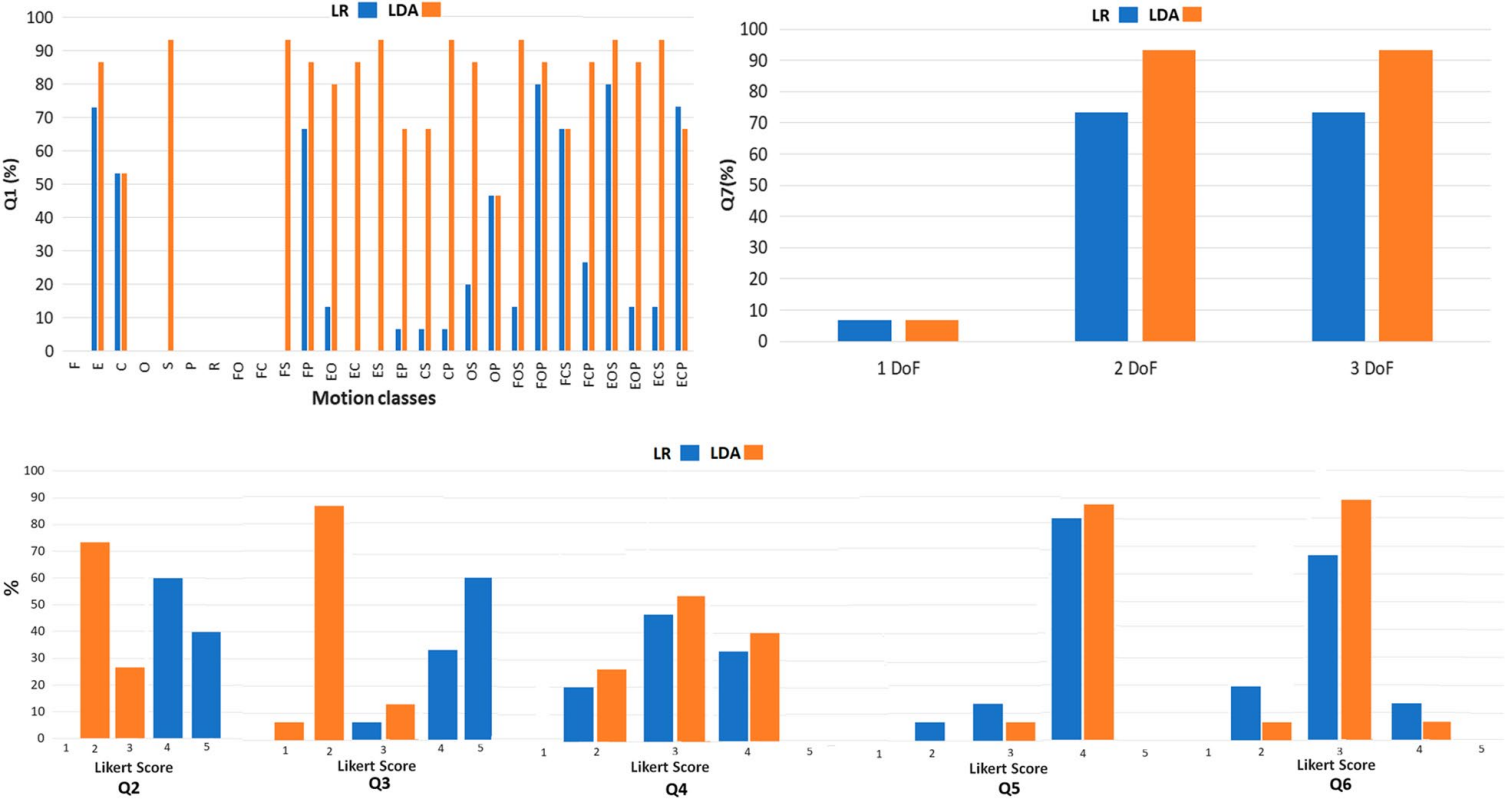
Results of the Likert questionnaire for both the LR (in blue) and LDA (in orange) algorithms

Thus, it is worth noting that the LR classifiers reached higher real-time performance, especially for the combined motions with wrist supination. This can be considered a positive result since previous studies have presented conflicting data regarding the contribution of the muscles involved in pronation and supination that are generally deep muscles [39].

The number of successful motions over the total number of motions attempted (27 motion classes x 2 repetitions= 54 total motion attempted) was reported as the completion rate (MCR) for both LDA and LR algorithms in Fig. 7. For the LDA algorithm, the MCR values were significantly lower than that obtained with LR for the following 2 and 3 DoFs motion classes: the MCR of the elbow flexion with wrist supination motion class was 24.12 % lower than that with the LR algorithm; the hand close with wrist pronation motion class (22.67 % lower); the elbow flexion with hand opening and the wrist supination class (20.00 % lower). These results confirm the lower robustness of LDA classifiers to classify in real-time the 2 and 3 DoFs combined motions with wrist supination.

A in-depth discussion can be carried out considering the MCT and MCR values related to the most important tasks for prosthetic users during daily living activities, reported in Table 1. According to [40], we reported the main activities needed for the personal independence of an adult. For instance, for the daily activity “brings a biscuit to your mouth”, the LR had the best values in terms of MCT ( $1.40 \pm 0.59$ s vs $2.45 \pm 2.06$ s) and MCR ($100 \pm 0$ vs $80 \pm 37$ ) with respect to the LDA. Also for the daily activity as “pour water into a glass”, the LR showed the lowest MCT ( $1.46 \pm 0.55$ s vs $2.08 \pm 1.53$ s) and the highest MCR ($100 \pm 0$ vs $87 \pm 30$ ). In addition, if we considered daily activities that involved the grasping task without wrist rotations as “bring something to your mouth”, and “Extend your open hand for giving something to someone”, the LR had the better values of MCT for both the tasks ($1.65 \pm 0.74$ vs $2.28 \pm 1.76$ and $2.89 \pm 2.01$ vs 2.84 ± 2.05 ) and MCR ( 94 ± 24 vs 80 ± 37 and $76 \pm 44$ vs $73 \pm 32$ ) than the LDA. These results obtained with the reported real-time performance metrics were confirmed also by the users’ answers to the questionnaire (Table 7). The better performance of the LR with respect to LDA can be considered an even more effective results due to the presence also of the wrist rotations in the considered daily living activities. The wrist rotations, in general, were more difficult to detect with superficial EMG electrodes because data regarding the contribution of the muscles involved in pronation and supination were generally deep muscles [39]. Finally, the LR-based parallel classification strategy seems to be very promising if we considered the importance for amputee subjects of controlling simultaneously more than two DoFs during daily living activities. Also the results obtained with the questionnaire confirmed that not all the motion tasks had the same level of difficulty, especially if we consider the 2 and 3 DoFs motion tasks.

The statistical analysis, based on the Mann–Whitney test, confirmed a statistically significant difference (“*”) between the LR and LDA MCT values (Fig. 8A) and between the LR and LDA MCR values (Fig. 8B) (at p < 0.05). A medium effect size was found for the mean MST values, while a large effect occurred for the mean MCR values (in Table 6). Thus, in real-time, the parallel classification strategy based on the three LR classifiers ensured better performance than the LDA classifiers and seems to be the most robust strategy.

## Comparison with other PR-based strategies

Traditionally, PR control strategies have provided significant improvements in extending the number of DoFs that can be controlled sequentially [41, 21].

However, the amputee’s ability to perform simultaneous movements in coordinated tasks can be further improved by considering more complex PR strategies, such as parallel classification.

In this study, three parallel LR classifiers were employed to simultaneously control multiple DoFs in a parallel classification strategy. Then, the offline and real-time performances were compared with the LDA algorithm.

Twenty-seven motion classes (up to 3 DoFs) were tested with both offline (in terms of F1Score) and online ( in terms of MCT, MST, MCR) performance measures. In literature, previous studies such as Young et al. [14] used a parallel classification scheme, based on three LDA classifiers to predict discrete and combined motions, considering two configurations: the 3 DoFs configuration consisted of fourteen motion classes in which six were discrete hand and wrist gestures (hand open/close, wrist supination/flexion, wrist flexion/extension) and eight were combined motion classes; the 4 DoFs configuration considered also the elbow joint and consisted of eight discrete motions (elbow flexion/extension, hand open/close, wrist supination/flexion, wrist flexion/extension) and twenty combined motion classes. Only the offline performance was reported for this study, and the classification errors were different for the two DoFs configurations: they ranged from 10.5 % ± 1.1 to 25.9 % ± 2.8 for the 3 DoFs configuration and from 9.5 % ± 2.2 to 29.3 % ± 2.8 for the 4 DoFs configuration. For both cases, the hand DoFs had the highest values of misclassification errors. The principal difference of this study from our strategy was the way of grouping the elbow, wrist, and hand motion classes. Indeed, we introduced a different 3 DoFs configuration, that included the elbow DoF instead of wrist flexion and extension, and considered in the same way the hand and wrist DoFs. With our strategy, we have demonstrated that both the LR and LDA algorithms provided good results, in terms of offline performance, for the 3 DoFs parallel classification strategy. Moreover, we have also demonstrated that the use of three LR classifiers ensured better performance than the LDA classifiers, especially for the combined motions with the wrist supination, and seems to be promising also for a reliable multi-DoFs control system (with misclassification error rates within 10 %, [4]).

In Young et al. [25] also the conditional parallel strategy was introduced in the three-DoFs configuration considering, as discrete motions, hand open/close and wrist flexion/extension, while, as combined motion classes, wrist extension with hand open, wrist extension with hand closed, wrist flexion with hand open and wrist flexion with hand closed. The results obtained for six healthy subjects have revealed, for the parallel conditional strategy, a reduced error rate equal to 6.6 % on discrete and 10.9 % on combined motions, compared to the parallel LDA strategy which had a much higher error rate (equal to 21 % on discrete and 20 % on combined motions). Also in [26], the LDA parallel classification strategy, in the 2 DoFs configuration, obtained an error rate equal to 18% to predict combined wrist/hand movements involving wrist rotation, wrist flexion/extension, and hand open/close in case of four able-bodied subjects.

With respect to the aforementioned studies ( [14, 25, 26]), limited by the lack of online performance measures, our parallel PR strategy was tested also in real-time: a new prediction was made every 90 ms, instead of 100 ms as in [28], with both the LR and LDA algorithms. This time saving of 10 ms can be exploited to have more predictions within 1 s (almost 12 predictions instead of 10) and, thus, can improve the robustness of the classification. Moreover, we have, also, demonstrated that the performance of the LR algorithm in terms of MCT values was better than that obtained with the LDA classifier, despite this last was considered the benchmark classifier for real-time employment [41, 42]. In the literature, other studies [22, 13] evaluated also the real-time performance metrics on four TMR patients for the control of a virtual prosthesis. However, Tkach et al. [22] adopted a different classification strategy based on a single LDA classifier that provided the simultaneous control of two DoFs for the following nine discrete and combined motion classes: no movement class, elbow flexion/extension, hand open/close, elbow flexion with hand open, elbow flexion with hand closed, elbow extension with hand open, elbow extension with hand closed. The use of a single classifier instead of multiple parallel classifiers working simultaneously for each DoF, required to train the single classifier considering each combined and discrete motion as a separate class. In our study, the use of the parallel classification strategy has the advantage of reducing the complexity of the three DoFs configuration, by grouping for each classifier all the discrete and combined classes of the related joint into only 3 output classes. Concerning the real-time performance metrics, [22] used the same parameters employed in this study, MCR and MCT, plus another different metric, named length of movement error; this last one is related to the use of virtual reality and it was defined as the percentage of the distance between the initial posture and the target posture.

In Young et al. [13] real-time results concerning four TMR amputee subjects (two transhumeral amputees and two shoulder disarticulation subjects) were presented. A single LDA classifier was used to simultaneously classify up 2 DoFs for a total of eight classes (four combined and four discrete); each discrete or combined motion was trained as a separate class as in [22].

Finally, with respect to the these studies [13, 22], our strategy was validated by using only healthy subjects. However, the promising results of our PR-based classification strategy, demonstrate the need for additional investigation into the benefits and practicality of using the parallel classification strategy to decode simultaneously complex motion classes that involving up to 3 DoFs. In particular, the possibility of controlling elbow movement with the simultaneous activation of hand and wrist DoFs is an important functional capability, especially for transhumeral and shoulder disarticulation amputees who have undergone TMR surgery [13].

## Conclusion

In the past, the simultaneous myoelectric control has been implemented by directly controlling multiple independent EMG sites [43].

However, pattern recognition control adds several benefits, such as the control of a greater number of DoFs, without the need for independent control sites, and a intuitive and more natural control of different joints [44]. In the previous myoelectric PR literature, few studies reported the application of simultaneous control of multiple DoFs with PR strategies [14, 25, 26].

To date, the single, hierarchical and parallel classification strategies, based on the LDA classifiers, were introduced to discriminate up to 19 wrist/hand gestures (in the 3-DoFs case), considering both combined and discrete motions [45].

In this study, a parallel classification strategy, based on three LR classifiers, was developed and tested to simultaneously discriminate up to 27 discrete and combined motion classes related to the elbow, wrist and hand joints (up to 3 DoFs).

The LR parallel classification strategy was tested on 15 healthy subjects by using 6 commercial sEMG sensors. Then, a comparative analysis among the performance of LR and LDA algorithms was done by using the Mann–Whitney test.

To this purpose, both offline and online analyses were taken into account for both LR and LDA classifiers to understand the robustness of the proposed algorithms for obtaining a simultaneous control. A feature set consisting of TD features, such as EMAV, EWL, ZC, SSC, RMS, VAR [32] was used to process the data with a window of 150 ms with an overlap of 100 ms [33].

All the three LR joint classifiers reached an average classification F1Score above the 90%.

The statistical analysis, based on the Mann–Whitney test, confirmed no statistically significant difference was found between the F1Score values of the LR and LDA “Elbow classifier”, “Wrist classifier”, and “Hand classifier”.

The offline results suggest that a parallel pattern recognition strategy based on three LR classifiers with TD features performs well for activating simultaneously different joints of a complex multi-DoFs prosthetic device.

In our study, both the LR and LDA classifiers were tested also in real-time and produced a new prediction every 90 ms instead of 100 ms, as in [28]. The same performance metrics, introduced in [28], were used, i.e. MST, MCT, MCR.

Specifically, the mean MCT values revealed that the difference in real-time prediction between the two algorithms is not marginal: for the 2 and 3 DoFs motion classes, the mean MCT was over 2 s for the LDA with respect to less than 2 s for the LR algorithm (Fig. 6). Moreover, also the MCR values were significantly lower for the LDA algorithm, especially for the combined motions employing the use of the wrist (Fig. 7).

The statistical analysis, based on the Mann–Whitney test, confirmed a statistically significant difference (“*”) between the LR and LDA MCT values (Fig. 8A) and between the LR and LDA MCR values (at p < 0.05). Thus, in real-time, the parallel classification strategy based on the three LR classifiers ensured better performance than the LDA classifiers and seems to be the most robust strategy, especially for the combined motions with the wrist supination. This result was confirmed also by the subjective user’s evaluation based on the Likert questionnaire.

In literature, the motion classes with wrist rotations are more difficult to discriminate because the contribution of the muscles involved in pronation and supination is generally associated with deep muscles [39]. Thus, the real-time robustness of the LR classifiers also for these complex motion classes, is a remarkable result.

The presented parallel LR classification strategy demonstrated higher classification performance both for the offline and real-time evaluation, with respect to the LDA based strategy.

The finding of this study provided valuable information to further improve with the use of LR parallel the simultaneous control of both multiple DoFs and discrete DoFs when desired by keeping low the number of sEMG electrodes used to discriminate different muscular patterns (only 6 sEMG sensors). The innovative organization of the DataSet into different TrainingSet has allowed each classifier to be trained with the examples of all available motion classes for each considered DoF. This type of DataSet organization is revealed to be a more robust training method than using motion specific data as separate classes.

The present performance evaluation has involved only healthy subjects, but future work will extend the use of the proposed PR based parallel classification strategy to persons with severe upper extremity amputations, such as shoulder disarticulation and transhumeral amputees with important implications on the functional status of the arm. It is planned to include in the study also TMR subjects.

Indeed, the PR strategy based on three parallel LR classifiers, can provide more life-like motions for amputees since it is able to predict simultaneously discrete and combined motions of up to three elbow, wrist and hand DoFs, instead of exclusively classifying sequential movements.

## Data Availability

Not applicable.
